# A Comprehensive Review on Methodologies Employed for Visual Evoked Potentials

**DOI:** 10.1155/2016/9852194

**Published:** 2016-02-29

**Authors:** Ruchi Kothari, Pradeep Bokariya, Smita Singh, Ramji Singh

**Affiliations:** ^1^Department of Physiology, Mahatma Gandhi Institute of Medical Sciences, Sevagram, Wardha, Maharashtra 442102, India; ^2^Department of Anatomy, Mahatma Gandhi Institute of Medical Sciences, Sevagram, Wardha, Maharashtra 442102, India; ^3^Department of Ophthalmology, Mahatma Gandhi Institute of Medical Sciences, Sevagram, Wardha, Maharashtra 442102, India; ^4^Department of Physiology, AIIMS, Patna, Bihar 801105, India

## Abstract

Visual information is fundamental to how we appreciate our environment and interact with others. The visual evoked potential (VEP) is among those evoked potentials that are the bioelectric signals generated in the striate and extrastriate cortex when the retina is stimulated with light which can be recorded from the scalp electrodes. In the current paper, we provide an overview of the various modalities, techniques, and methodologies which have been employed for visual evoked potentials over the years. In the first part of the paper, we cast a cursory glance on the historical aspect of evoked potentials. Then the growing clinical significance and advantages of VEPs in clinical disorders have been briefly described, followed by the discussion on the earlier and currently available methods for VEPs based on the studies in the past and recent times. Next, we mention the standards and protocols laid down by the authorized agencies. We then summarize the recently developed techniques for VEP. In the concluding section, we lay down prospective research directives related to fundamental and applied aspects of VEPs as well as offering perspectives for further research to stimulate inquiry into the role of visual evoked potentials in visual processing impairment related disorders.

## 1. Introduction

Among the recently advancing neurologic diagnostic tools, the role of evoked potential studies has been in flux during the past few decades. The evoked potentials (EPs) are bioelectric signals produced by the central nervous system when it is triggered by an explicit external event. In the last five decades, evoked potentials have evolved from an exigent technological tool to routinely utilized investigation in the field of clinical neurology. Eventually recording the spontaneous electrical activity of the brain from electrodes overlying the scalp has become a clinical practice for the last so many decades.

*Historical Background*. Several forms of electrodiagnosis used in neurology have much shorter histories than electrotherapy, for they have their basis in fundamental neurophysiology rather than in quasiquackery [1].

In the period under review, only electroencephalography, electromyography, the action potential of nerve, and the evoked cortical potential have a history. This history which they largely share essentially stems from discoveries of Galvani [2] who in his well known “Commentarius” first published his claims for intrinsic animal electricity which was the culmination of many years of experimentation. He made three chief observations in frog's nerve-muscle preparation:

- It twitches when touched by an observer.
- The atmospheric electricity of a thunder storm could be used to stimulate the frog's legs if a long wire was stretched across the roof (the principle of lightning conductor).
- The frog's legs twitched when hung by hooks from railing of house even in the absence of a thunderstorm.

Du Bois-Reymond [3] and Hermann [4] demonstrated normal electric potentials recorded from the surface of a muscle on contraction. Du Bois-Reymond experimented this in human muscles and gave birth to electromyography. He identified the nervous principle with electricity.

Caton [5] discovered the cerebral counter parts and found not only the electroencephalogram but also the evoked potential changes on sensory stimulation especially with visual stimuli. He provided for the first time the method for mapping the localisation of sensory areas in cortex supplanting the crude ablation techniques till then. He demonstrated the brain waves of his animals by optical magnifications of the movements of the meniscus in his Thompson's galvanometer. In 1887, he reported his experiments on 45 animals describing his operating techniques, electrodes, and instrumentation.

Beck [6] marked the position of electrodes that gave him response to light and faint response to sound.

Pravdich-Neminsky [7] gave the first photograph of an evoked potential recorded of the cortex of a dog on stimulation of the sciatic nerve.

Berger [8] named spontaneous ongoing activity of the brain as “Das Elektrenkephalogram” and in 1940 launched the electroencephalogram as clinical neurologic test which is now used worldwide. He acknowledged the previous works of Caton.

The responses elicited by visual stimuli were recorded first in animals right from the surface of the pia mater in the 1930. It was recognized then that the alpha rhythm observed in normal electroencephalographic traces could be enhanced by exposure of the eyes to a flashing light at same frequency. If the eyes get exposed to repetitive flashes of changing frequencies, the electrical changes produced from the scalp electrodes become diminutive and more or less get vanished against the backdrop of the usual spontaneous activity of the brain.

The problem of identifying these minute electrical signals has been largely taken care of by the launch of averaging techniques. When there is averaging of the responses to a multitude of similar visual stimuli, the discrimination from irrelevant cortical events can be greatly enhanced. From the applied point of view, the advent of signal averaging is a crucial development as it allows recording of VEPs as tiny as 2 to 3 *μ*V. Moreover the kind of response, its waveform pattern, and amplitude can be associated with the type of visual stimulus in truly astounding manner [9].

## 2. Clinical Significance and Advantages of VEPs 

The visual evoked potential (VEP) is among those evoked signals that can be picked up by the electrodes overlying the scalp region. Normal visual evoked response is acquired if there is no lesion in the entire visual pathway but breaches anywhere in the visual system can generate abnormal VEPs. Visual evoked potentials have been clinically available for more than three decades. VEP averages occipital lobe activity evoked from contrast stimulation of the visual system. Despite advances in imaging such as MRI, it remains a valuable tool to document occult lesions of the central visual channels especially within the optic nerve.

Visual evoked potentials have proved to be useful in

- testing visual sensory function when clinical examination is not reliable,
- investigating purely subjective symptoms and detecting whether they have an organic origin,
- better assessing the causative mechanism of neurologic deficits and functional recovery,
- monitoring cerebral functions when the patient's condition is critical and at risk in the operating theatre or during intensive care [10].

An abnormal visual evoked potential in clinical cases may furnish objective evidence to an unsuspected or suspected but not proven abnormality. It allows one to quantify and objectively follow up a known lesion [11]. It provides a means to assess the functional integrity of the visual pathway whereas imaging techniques such as MRI evaluate mostly their anatomical and structural basis. One of the most remarkable properties of VEPs is their resistance to anaesthesia and sedative drugs and in comparison with EEG activity even damage of the cerebral hemispheres [12]. This permits their use for monitoring the functionality of the visual pathways in situations that render EEG useless.

First introduced by Adrian and Matthews [13] VEP has been in use for clinical and research purposes since more than five decades. When Halliday et al. [14] clinically utilized pattern reversal VEPs in the diagnosis of patients of optic neuritis, it gave way to VEPs for being particularly used in the assessment of cases having suspected multiple sclerosis (MS).

Pattern induced VEPs are more sensitive to optic nerve lesions than flash evoked responses [15]. This commonly used pattern reversal method for VEP stimulation was developed and popularized in the early 1960s [16].

Diseases of the optic nerve in man may affect a part or all of the optic nerve fibers. The resulting symptoms are quantified by measurements of visual fields, visual acuity or color vision tests. Pattern evoked visual responses have become a reliable tool to recognize lesions in the optic pathway. This method is so sensitive that demyelinated plaques can be recognized even in the absence of any clinical symptoms as measured by perimetry, visual acuity, or color tests [17, 18]. Thus VEPs may identify visual malfunction in patients with complaints in vision but with no objective findings on examination and in patients lacking visual symptoms [19].

## 3. Standards and Protocols

There had been no reference protocols for VEP recording or analysis for quite a long period of time. Several diagnostic clinics and laboratories measured VEPs using incongruent techniques, and subsequently, it resulted in many conflicting and inconsistent estimations of parameters of the VEP, raised skepticism regarding the scientific utility of the technique, and the anomalies in relation to particular disorders.

To make clinical care better and permit comparison different laboratories, it is recommended that a standardized methodology should be used. For this purpose, the International Federation of Clinical Neurophysiology (IFCN) [20] Committee and International Society for Clinical Electrophysiology of Vision (ISCEV) [21] have put forth their Recommendations and Guidelines which present minimum protocols and requirements for fundamental VEP recording in a clinical set-up.

In this standard, three stimulus protocols have been laid down.

A subset of stimulus and recording conditions has been chosen by ISCEV that impart fundamental clinical information and can be executed in most clinical electrophysiology units across the globe. The waveform of a VEP depends upon the temporal frequency of the stimulus. At low temporal frequencies, that is, when stimuli are recurring in less than twice or thrice per second, the waveform appears as a number of discrete excursions and is called transient VEP. All the VEPs mentioned in ISCEV standard are of transient type.

## 4. Methods & Modalities of VEPs 

Majorly two kinds of visual stimuli are used to generate VEPs:

*(a) Unpatterned Flashing Lights*. Brief flashes of light with no perceptible pattern or contour comprise the* unpatterned stimulus*. Such simple unpatterned VEPs are of use when pattern stimulation is rendered inappropriate in cases of poor optical media, lack of cooperation, or diminished vision.

*(b) Patterned Stimuli*. The recommended patterned stimulus is a checkerboard with black and white pattern. All the checks have to be square making an equal number of light and dark ones. Patterned stimuli are defined by a visual angle subtended by the side of a single check in degrees (°) or minutes of arc (min) subtended at the eye. One degree equals 60 min of arc. The size of the individual checks usually reported in terms of visual angle in minutes of arc.

Pattern stimuli can be presented in three ways:

(1) *Flash VEP* can be generated by a pattern of flashing luminance spanning a visual field of about 20 degrees. It can be accomplished by making use of a flashing screen, a stroboscopic light that can be held in hand or by placing in front of the patient a Ganzfeld bowl. The flash rate has to be kept as 1.0 Hz ± 10%, that is, 1/sec.
 The flash VEP waveform consists of a succession of negative and positive waves. The first distinguishable wave appears 30 ms after the stimulus and the latter components are obtained up till 300 ms. Peaks are consequently labelled in a numerical series as negative and positive. Such a nomenclature enables differentiation of flash VEP from the pattern reversal type. Mainly the significant components of the flash VEP evident are the N2 and P2 peaks. P2 amplitude is to be measured from the P2 peak at approximately 120 ms to the former N2 negative peak at about 90 ms.
 Flash VEP is of particular use for patients who are incapable or show reluctance for pattern VEPs and when pattern stimuli seem invalid due to the presence of opacities in media.
(2) * Pattern onset/offset VEPs* can be produced by pattern stimulation of small 0.25° (15 min) and larger 1° (60 min) checks.
 In this case an abrupt exchange of checkerboard pattern occurs with a diffuse gray background. Pattern onset should last for 200 ms followed by 400 ms of diffuse background.
 In adults, pattern onset/offset stimulation elicits VEPs comprising of three prominent peaks: a positive C1 wave at about 75 ms, a negative C2 wave at about 125 ms, and lastly a positive C3 wave at around 150 ms. Amplitudes are always estimated from the peak of preceding wave.
 The pattern onset/offset stimulus fits best for identifying or verifying cases of malingering and for patients having nystagmus because the technique is not much perceptive to confounding factors like deprived fixation, eye movements, or intentional defocus.
(3) *Pattern reversal VEPs* can be produced by checkerboard stimulation generating larger 1-degree square checks with visual angle of 60 min of arc and smaller square checks of 0.25 degrees, that is, 15 min of arc. There is alternation of these checks from black/white to white/black without change in the overall luminance of the screen at a specific reversal rate. For most clinical purposes, pattern reversal is the optimum stimulus. Pattern reversal VEP waveforms are more consistent with regard to morphology and timing as compared to VEPs produced by other stimuli.
 The pattern reversal VEP waveform comprises of N70, P100, and N155 peaks. Their peaks are labelled as positive and negative succeeded by their latency values. As per the standard, the P100 amplitude is estimated from the peak of preceding N70 wave. P100 wave is the most robust peak with comparatively minimal interindividual variability, nominal within-subject intereye difference, and negligible variation with high repeatability.

**Figure 1:**
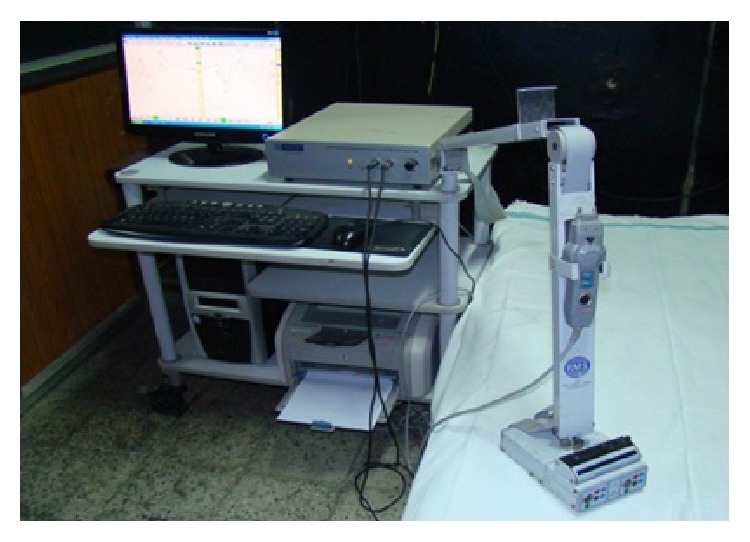
Evoked Potential Recorder (RMS EMG EP MARK II).

**Figure 2:**
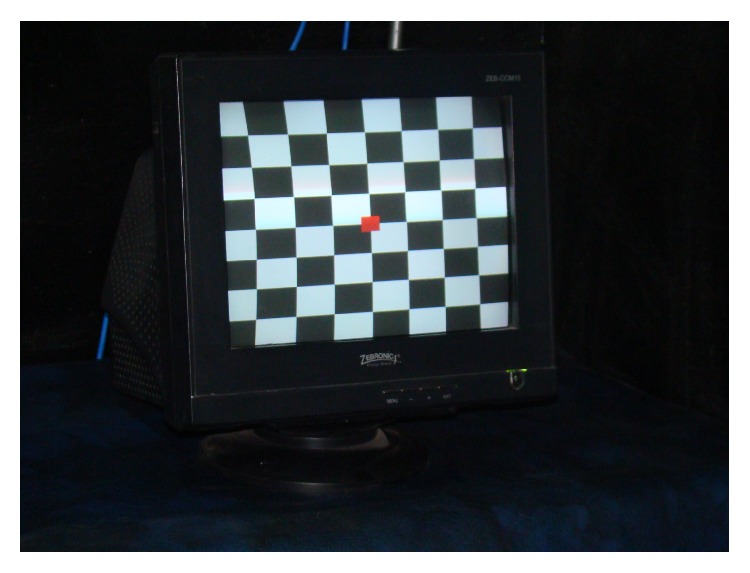
VEP Monitor displaying 8 × 8 checker board pattern.

PRVEPs with full-field stimulation are useful in assessing the integrity of anterior visual pathways. The P100 latency prolongation in one eye is typically indicative of optic nerve or prechiasmal dysfunction [22]. The probable use of PRVEP in diagnosing and monitoring progression of open angle glaucoma and its association with Mean Defect and Pattern Standard Deviation of Humphrey Visual Field has been recently evaluated [23–25]. In these studies, VEPs were recorded using transient pattern reversal stimulation where a checker board with black and white pattern was produced as a full-field on a VEP Monitor by an Evoked Potential Recorder (RMS EMG.EP MARK II) having inbuilt electronic pattern regenerator (Figures 1 and 2).

The most commonly employed pattern VEP stimuli are gratings and checkerboard patterns (Figure 2).

Sinusoidal gratings on the other hand have progressively emerging and vanishing borders and can be illustrated by a single-frequency (fundamental) sinusoidal function with power along one axis only.

Trivial defects in the visual system that are missed with checkerboard pattern VEP can be sensitively quantified by pattern VEP technique with sine wave gratings oriented at various visual angles.

## 5. Stimulator

TV or video monitor an oscilloscope or on a rear projection screen using a projector and a movable mirror can be used as different modes for exhibiting pattern stimuli. As such no stimulator is perfect. Equivalent physical characteristics of the stimuli can only give analogous results among laboratories.

## 6. Electrodes

The skin electrodes ideally should make reasonable contact, be neutral electrically, and have a minimal electrical resistance. The most widely used and customary ones are silver coated silver chloride disc electrodes. They are positioned on the scalp region after proper skin preparation by cleansing, degreasing, abrading, and applying a conducting jelly or electrode paste and gently onto the area using a cotton swab for ensuring satisfactory and steady electrical connection. Practically, the selection of electrode location depends on precise feature of the VEP being explored. The VEP protocols of ISCEV standard are circumscribed for a single recording channel with active electrode on a midline occipital position. A three-channel montage, using the midline and two lateral active electrodes can be utilized if chiasmal or retrochiasmal disease is expected.

### 6.1. Electrode Placement for PRVEP

**Figure 3:**
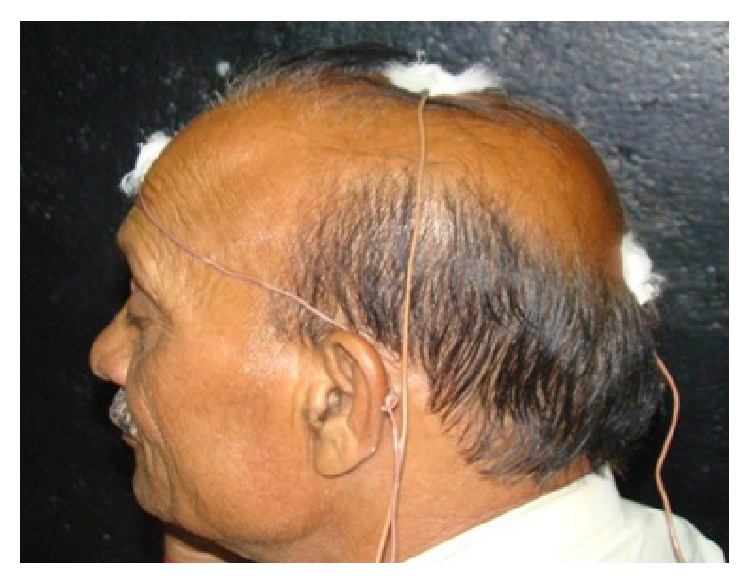
VEP Electrode Placements on a subject as per 10–20 International System.

Electrode Placement is kept in accordance with* 10–20 International System* [26] as mentioned below:

- Reference electrode (Fz) placed at the forehead.
- Ground electrode (Cz) at the vertex.
- Active electrode (Oz) at approximately 2 cm above the inion (refer Figure 3).

Maximum amplitude is generated by this montage usually and waveform produced is mostly artifact free, the reason being the maximal macular projection the occipital pole.

## 7. Setting for Pattern Reversal VEP Recording Generated by Checkerboard Stimulus Configuration

The typical recording arrangement for pattern reversal visual evoked potentials generated by Checkerboard Stimulus Configuration includes the following:

- The patient is made to sit at a suitable distance from the visual stimulus bearing the appropriate refraction (corrected for the test distance).
- The recording (active) electrode is placed on the posterior scalp and joined to the positive input of the differential amplifier.
- A reference electrode is placed on a visually neutral site on the head like the earlobe and connected to the negative terminus.
- A ground electrode is placed on the forehead.
- A stimulus generator is employed to select the required type of stimulus (flash, pattern reversal, or pattern onset-offset), temporal frequency, type of pattern (checks or grating), and magnitude of the pattern elements. The stimulus generator transmits an impulse to the signal averaging computer which is synchronous with the presentation of stimulus and elicits each averaging epoch.
- The signal averaging computer usually regulates the duration of the averaging epoch, the number of averages accumulated, the filtering of the signal and signal analysis.
- Responses to about 200 stimuli (100–300) are amplified and averaged for each eye, which are then analyzed by inline computer having automatic artifact rejection mechanism.
- Monocular recording is performed separately for the left and right eyes.
- A minimum of two trials for each eye are usually procured and then superimposed for ensuring replicability of the VEP pattern.

## 8. Recommended Recording Stimulus Parameters (IFCN and ISCEV)

The parameters are as follows:

- Sensitivity *⇨* 2 *μ*V.
- Rate of presentation/sec *⇨* 2 (1.8–2.2) reversals (transient VEP), 4–8 Hz (steady state).
- Amplification *⇨* 20,000–50,000 times.
- Electrode impedance *⇨* 5 kilo ohms.
- Filters *⇨* high pass and low pass filters *⇨*  <2 Hz and at >100 Hz.
- Averaging *⇨* number of sweeps (Epochs) per average *⇨* 200.
- Analysis time (Sweep duration) *⇨* 250–300 ms.
- Mean luminance of pattern between the centre and periphery of the field *⇨* 50 cd m^−2^ (40–60 cd m^−2^) and it should be constant.
- Background luminance *⇨* 20–40 cd/m^2^.
- Contrast *⇨* between 50 and 80%.

ISCEV standard does not encompass some specific and expanded VEP protocols. They are as follows.

### 8.1. Steady State VEP

The VEP waveform is largely determined by the temporal frequency of the stimulus. If the stimulation rate is faster, that is, more than about 3.5/sec, then it tends to produce a waveform which is more or less sinusoidal and is known as “steady state.” It can be analyzed by fast Fourier transform which provides phase and power values for the waveform. This type of VEP using a rapidly repeated pattern has its utility in the assessment of visual field and acuity of infants. It might a better approach as per some current testimony [9].

### 8.2. Sweep VEP

Many neurophysiology laboratories and clinics have adopted sweep VEP for acuity testing in recent years. This is particularly useful for the examination of nonverbal children and of malingering patients. For this purpose, a pattern stimulus that is alternated at a high temporal frequency rate (in the range of 5 to 15 Hz), producing a steady state visual, evoked response. A discrete Fourier transform (DFT) is performed on the recorded signals and provides a real time measurement of the amplitude and phase of the response. In order to measure visual acuity, the size of the pattern is reduced rapidly. Within 10 seconds, 20 different pattern sizes are presented in succession. This sweep of the spatial resolution domain allows an estimation of visual acuity from the smallest pattern size producing a response [27].

#### 8.2.1. Methodology

The program as developed by Metrovision production group [28] starts by creating a picture of a cartoon to capture the attention of the child. The presentation of a checkerboard with large dimensions follows the picture of cartoon. The frequency spectrum of the recorded signal is displayed in real time on the control monitor and allows the operator to visualize the response that is characterized by a peak at the stimulation frequency. a rapid series of 20 different patterns of diminishing sizes is generated by sweep stimulations that are triggered by an operator.

### 8.3. Motion VEP

A thorough examination of the magnocellular and dorsal pathway of motion processing visual is a vital augmentation of VEP.

#### 8.3.1. Motion-Onset VEPs

Motion-onset VEPs exhibit maximum amplitudes and minimal inter- and intraindividual variability. As early or selective involvement of the magnocellular and dorsal system is suspected in some neurological disorders, the usefulness of motion-onset VEPs is suggested as part of the electrophysiological central nervous system assessment. Furthermore, this technique may outdo other methods in identifying motion processing involvement. Such VEPs may enhance the sensitivity of this test in diagnosing CNS disorders including multiple sclerosis, encephalopathies, dyslexia, and glaucoma. Hence, this modality of VEP appears to offer maximum potential to be useful for diagnostic purpose [29].

Classically, the motion-onset VEPs comprise three major peaks—P1, N2, and P2. Two forms of the motion-onset VEP shape are there which differ in their preponderance of either a motion-specific N2 peak or pattern-specific P1 peak. The P1 peak mostly is indicative of the pattern-related function of the parvocellular pathway. The N2 peak possibly depicts the motion processing system activity and appears to emerge from the extrastriate temporooccipital and associate parietal cortical areas and preponderates usually in the right hemisphere.

#### 8.3.2. Motion-Reversal VEPs

They manifest the responses to variations in the direction of motion.

#### 8.3.3. Motion-Offset VEPs

Such VEPS tend to include pattern-related (most likely pattern-on) constituents due to prolonged duration of motion because the motion-processing visual cortex adapts.

#### 8.3.4. Chromatic Moving Stimuli

Certain stimulus parameters recently proposed for induction of the visual motion processing system and for appearance of the motion-onset specific N2 peak (with latency of 160–200 ms) from the extrastriate temporooccipital and/or parietal cortex include

- low luminance (20 cd/m^2^),
- low contrast (10%—sinusoidally modulated) of a moving structure,
- low velocity,
- low temporal frequency (<ca. 6 Hz).

A brief duration of motion (about 200 ms) and a long interstimulus gap of at least 1 sec. diminish motion adaptation and preponderance of a pattern-related P1 peak. In comparison to a unidirectional translation, radial motion (with increasing velocity and decreasing spatial frequency towards the periphery) elicits greater responses.

Even so, the problem of generating the stimulation and the employment of diverse recording and evaluation methods has hampered the inclusion of motion-related VEPs in routine neuroophthalmological diagnostics [29].

### 8.4. Chromatic (Color) VEP

The alteration in color pattern reversal visual evoked potential (CPR-VEP) in patients of primary glaucoma has been studied using various temporal frequencies with different color stimulations (black/white, red/green, and blue/yellow) by Wang et al. [30] and Tong et al. [31] who obtained CPR-VEP using Vision Monitor visual electrophysiograph. They reported extended P100 wave latencies with the increase of temporal frequency with different color stimulations (black/white > blue/yellow > red/green). The CPR-VEP P100 amplitude was reduced in their glaucoma group while temporal frequency was increased under three color stimulations. The authors concluded that the changes of CPR-VEP P100 amplitude can objectively reflect the glaucoma visual function damage.

### 8.5. Binocular (Dichoptic) VEP

Using this new VEP technique, unambiguous objective evidence of cortical binocularity can be recorded. The cortical interactions at the difference frequency (beat) elicit binocular VEPs. Dichoptic VEP can be performed quickly and easily on young children and gives a quantitative assessment of cortical binocularity that may not be determinable by standard clinical methods. This technique may also prove useful for the preoperative gradation of binocular potential and prediction of postoperative binocular fusion [32].

#### 8.5.1. Methodology

Each eye is presented with separate visual stimuli by alternating field stereoscopy of the system. By this system, alternation of the binocular image pairs to the right and left eyes occurs at a high rate on a single video monitor. Spectacles incorporating light-scattering liquid crystal lenses are worn by the subject and the lenses alternate electronically between opaque and clear modes in synchrony with the monitor. For identification of cortical binocularity, VEP activity is analyzed mathematically by the system and significant responses at test frequencies indicating binocular cortical interactions exclusively are detected.

Binocular stimuli of three kinds are presented:

1. *Dynamic random dot correlograms*: these correlograms are formed only when moving random dot patterns that are offered to each eye oscillating between two phases, correlated and anticorrelated [33].
2. *Dynamic random dot stereograms*: for the stereograms, segments of random dot patterns delivered to each eye shift horizontally relative to each other at a predetermined rate, alternately creating crossed and uncrossed binocular discrepancies [33].
3. *Dichoptic checkerboard stimuli*: these are the conventional checkerboard patterns that reverse at various frequencies (rates) for each eye [33].

### 8.6. Multichannel VEP

Meticulous concern is required for interpretation of multichannel PRVEPs due to the concept of paradoxical lateralization. When a large field, large check reversal stimulus and common reference recording to Fz is employed then this phenomenon occurs, whereby response recorded at a lateral scalp location is generated by activity in the contralateral hemisphere of the brain. Multichannel recording is obligatory for the accurate diagnosis of dysfunction of intracranial visual pathway. There occurs an asymmetrical distribution of the VEP over the posterior scalp with dysfunction at, or posterior to, the optic chiasm, or in the presence of chiasmal misrouting (e.g., in ocular albinism).

- *Chiasmal dysfunction* produces a “crossed” asymmetry in which the lateral asymmetry acquired on stimulation of one eye is reversed when the other eye is stimulated.
- *Retrochiasmal dysfunction* presents with an “uncrossed” asymmetry whereby the VEPs obtained by stimulation of each eye exhibit a comparable asymmetrical distribution across the hemispheres.

For multichannel VEP, the recommended pattern stimulus is presentation of field of 30°. A minimum of two channels and at least three active electrodes, two of the lateral ones located at O1 and O2, and a third midline active one at Oz is required for detection of lateral asymmetries. The reference for all the active electrodes should be Fz. Ancillary electrodes at PO7 and PO8 also referred to Fz may augment the sensitivity to lateral asymmetries [21].

### 8.7. Hemifield VEP

The P100 waveform recorded from inion is algebraic sum of individual half-field VEPs. On hemifield stimulation, the ipsilateral visual cortex reveals the positivity whereas on the contralateral side and a negativity is recorded. The majority of P100 is generated by the lower half of the central field and the upper visual field may contribute as negative peak at frontal location [34]. The sensitivity of half-field VEP testing is better than full-field testing in identifying lesions of the visual system at chiasmal or postchiasmal sites. The cause of ambiguous findings of full-field testing can often be clarified by this technique. Hemifield stimulation may reveal lesion in visual pathway in spite of normal full-field PVEPs. This test needs more of patient cooperation and is technically more taxing than the full-field. Thus hemifield VEPs are not yet considered by IFCN [20] sufficiently reliable and sensitive to be recruited for the appraisal of retrochiasmatic dysfunction.

### 8.8. Multifocal VEP

A new technique which has shown promise in the study of the visual function is the multifocal VEP (mfVEP). The visual evoked potentials (VEPs) can be recorded simultaneously from many areas of the visual field in a matter of minutes with the multifocal technique. The patient has to look at a checkerboard display having 60 sectors. Each of the 60 sectors of the display is an independent stimulus with 16 checks, eight black and eight white [35, 36].

The mfVEP cannot be considered as a smaller version of conventional VEP. mfVEP responses may differ among individuals in terms of the amplitude and waveform as well as across the visual field within an individual. These dissimilarities are associated with cortical anatomy, and with the cortical sources contributing to the mfVEP. V1 is the predominant generator site for mfVEP. For the analysis of the responses and for displaying the results, a special approach is followed. A map of the defects is generated in this method by comparing the monocular responses from the two eyes of an individual. This map is in the form of a probability plot similar to the one used to display visual field defects measured with automated perimetry [35, 36].

The multifocal visual evoked potential (mfVEP) technique has gathered considerable attention from researchers in recent times. They are considered the best in evaluation of asymmetry of visual function brought about by any optic nerve dysfunction.

This type of VEP has been used for excluding functional visual loss, in the diagnosis and follow-up of patients with optic neuritis/multiple sclerosis and monitoring progression of the disease [37]. A pathology which failed to be spotted with a conventional VEP can sometimes be picked up by this modality.

### 8.9. LED Goggle VEP

**Figure 4:**
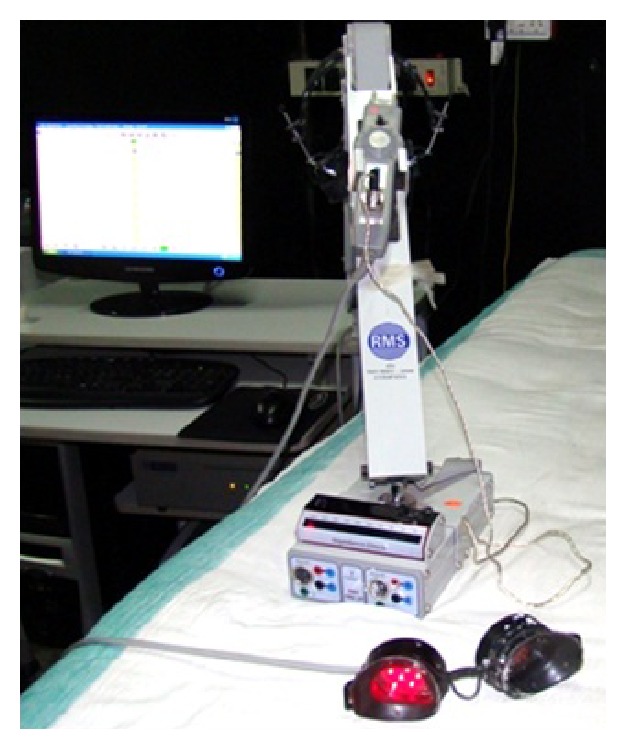
Typical setting for Goggle VEP.

Another technique for visual stimulation involves the use of a matrix of light emitting diodes (LEDs). The LED elements can be placed in goggles and arrayed in a pattern or a grid (refer Figure 4). A display board made of light emitting diode can be viewed from a distance or otherwise LED goggles can be kept directly over the eyes and taped gently in place. There is an added advantage of goggles that they provide a very large field of stimulation that reduces the effect of changes in direction of gaze to a minimum. But then they have the limitation that stimulation usually takes place through closed eyelids and so the eyes cannot be monitored. LEDs can come in various colors but the most commonly used is red [38]. The intensity of LED stimulus is not great enough to bleach the visual pigment and there is no photochemical effect at the recording electrodes. This method is gaining popularity particularly in the nursery where it is not disruptive to the other infants and can be performed with the infant lying comfortably in the incubator or warmer [39]. The utility of LED goggles in obtaining Flash VEPs in children with delayed milestones and with Spastic Cerebral Palsy has been documented in recent studies performed at our laboratory [40, 41]. A typical setting for Goggle VEP conducted here is provided in Figure 4.

## 9. Scope for Future Research

The quoted accounts of diagnostic applications of different modalities of VEPs provide fairly encouraging results; however, these results should be confirmed by various laboratories. Moreover, it is essential to gather information regarding the specificity of VEPs. Validation of diagnostic value of VEPs by assessing their specificity in neurophysiological labs is mandatory before their application as routine examinations. Despite the fact that the specificity of any sort of VEPs is quite petite, their prospective and role in differential diagnostics can be considerable if their findings correlate with patient's anamnesis. The amalgamation of various forms of VEPs could enhance their sensitivity and specificity to pathological findings.

## 10. Future Prospects of VEPs 

The major use of VEPs is in the detection of subclinical lesions within the visual system; asymptomatic optic neuritis could be easily detected and its presence may aid in the diagnosis of multiple sclerosis. Abnormalities of optic nerves are poorly visualized by MRI making VEPs an important adjunct when diagnosis of demyelinating disease is in doubt. VEPs can also help discriminate blindness from hysteria and malingering. If a patient reports visual loss, a normal VEP strongly favours a psychogenic disorder. VEPs could be extremely useful not only for the clinical neurophysiologist or ophthalmologist but also for neurologists and neurosurgeons since many of the neurological disorders present with visual abnormalities [4]. This test complements the subjective test of visual function by providing “that” small piece of extra evidence that may sometimes be decisive in reaching a firm diagnosis.

## 11. Concluding Comments

By this comprehensive review we conclude that VEP recordings can be performed by multiple kinds of procedures to assess the integrity and function of visual pathway. The selection of particular VEP stimuli and protocol to be applied depends on the symptoms, anamnesis and other available information of the patient. The VEP testing is a promising methodology that may permit understanding the neural processing of the evoked biologic signals in a broader and yet in a lucid manner.
